# Procedural justice and forensic mental health: An introduction and future directions

**DOI:** 10.1177/00258024231206865

**Published:** 2023-10-17

**Authors:** Jack Tomlin, Sarah Markham, Ciska Wittouck, Alexander Simpson

**Affiliations:** 1 School of Law and Criminology, University of Greenwich, UK; 2 Department of Biostatistics and Health Informatics, Institute of Psychiatry, Psychology and Neuroscience, King's College, London, UK; 3 Department of Criminology, Criminal Law and Social Law, Institute for International Research on Criminal Policy, 26656 Ghent University, Belgium; 4 Complex Care and Recovery Program – Forensic Division, Centre for Addiction and Mental Health, Toronto, ON, Canada; 5 Department of Psychiatry, 7938 University of Toronto, ON, Canada

**Keywords:** Procedural justice, forensic mental health, patient engagement, autonomy, shared decision-making

## Abstract

This article advocates for integrating procedural justice principles into forensic mental health services to enhance patient engagement and autonomy. Procedural justice, broadly defined as fair decision-making processes, is introduced and key principles including voice, neutrality, respect and trustworthiness are described. Evidence suggestive of positive outcomes following procedural justice experiences, such as improved satisfaction, collaboration and reduced perceptions of coercion is outlined. Practical applications are suggested, including staff training and reflective practices using procedural justice principles. The article then calls for further research to explore patients’ and staff members’ experiences of procedural justice in forensic settings, develop measurement tools, undertake intervention studies and establish causal links between procedural justice and outcomes important for forensic patients.

## Introduction

Forensic mental health services are increasingly considering models of care that embrace patient autonomy, choice and engagement in care.^1^ This can be seen in recent work on shared risk assessment, recovery colleges and efforts to involve patients in research.^2,3^ Involvement in decision-making has the potential to improve therapeutic relationships, increase satisfaction with care and support personal recovery outcomes.^4^

Research shows that (physical and mental health) patients want such involvement: a review of 115 studies of shared decision-making found that most patients in studies published since 2000 preferred shared decision-making roles.^5^ A recent review of the forensic mental health literature found that patient disempowerment was a frequently cited barrier to recovery and that patients often felt inadequately informed about the goals of their care.^6^ Such exclusion has negative effects on treatment, motivation to participate in care, and quality of life, and has been linked with aggressive behaviour, hostage taking and absconding.^1,7,8^

One of the key tensions in implementing autonomy- and choice-promoting approaches in forensic settings is that decisions are often made *for* patients, instead of *with* or *by* them. This might be for legal, clinical, resourcing or safety reasons and can sometimes be justified. However, the specific medico-legal nature of forensic mental health services poses challenges to autonomy- and choice-promoting approaches that are not present in other areas of healthcare. These include restrictions on leave into the community, accessing hospital grounds, receiving visitors, selecting meals or withdrawing consent for treatment. It is therefore worth exploring ways of improving patient engagement in care, and specifically, decision-making, within these constraints.

Procedural justice is a conceptual and theoretical framework that can help us understand how to maximise patient involvement in shared decision-making, as well as experiences of disenfranchisement from decision-making, its consequences and what can be done to improve outcomes where difficult decisions need to be made *by*, *with* or *for* patients. This article argues that procedural justice should be a core concept in forensic mental health care. When viewed through a human rights perspective, it can help reduce coercion and promote agency. We explore definitions of procedural justice, briefly describe the supporting evidence, outline why procedural justice experiences matter from a social-psychological perspective, examine its value in staff–patient relationships, and touch upon interventions to train staff to use procedural justice principles.

## Defining procedural justice

Procedural justice can be broadly defined as ‘as the fairness of processes used by those in positions of authority to reach specific outcomes or decisions’.^9^ Thibaut and Walker^10^ conducted one of the earliest studies on the topic. They asked participants in a mock dispute resolution trial what made proceedings feel fair. Appraisals of fairness cited *process* and *outcome* control as important. Participants highlighted the role of representation, that is, that people – directly or through advocates – could tell their side of the story. This began a rich line of research that has, in most contexts but not all, found that people involved in legal proceedings view the way they are treated by authorities and the procedures used as equally or more important than the outcomes of proceedings.^11^ This has been reported for some victims of crime, where the outcome of criminal proceedings (e.g. prosecution of the perpetrator) was a weaker predictor of overall satisfaction than perceived treatment by the police.^12^

So, what do procedurally just experiences look like? Current research has coalesced around four key principles that are fundamental to a person's experience of procedural justice.^13^ *Voice*: people have the chance to present their side and experiences and be listened to. Importantly, these views must be taken seriously and incorporated into decision-making; otherwise, this can lead to a so-called ‘frustration effect’, which is detrimental to trust.^14^ *Neutrality*: people perceive authority figures as unbiased, applying understandable and transparent rules consistently and using clear evidence. This evidence needs to be accessible to patients; this closely reflects the clinical understanding of ‘informed consent’.^15^ *Respect and dignity*: individuals feel treated courteously as unique and valuable persons with rights and needs respected. *Trustworthiness*: authority figures are perceived to be working in an individual's best interests and are considered authentic and sincere. Some academic work extends this framework, adding *information* (explicitly recognising the role of clear and accessibly communicated information), *performance* (competency of staff/power holders) and *authoritativeness* (e.g. compromising, being ‘firm but fair’).^16–18^

Though these principles have received broad empirical support, the extent to which these are valued will vary between individuals and across time and context.^19^ What people care about most when evaluating the procedural fairness of an interaction will be shaped by: political and moral views, neighbourhood norms, experiences of systematic distributive injustice, the nature of the current encounter with legal authorities (e.g. in a policing context: calls for assistance vs. arrests and searches), whether an encounter involves high stakes and experiences of underenforcement of antisocial behaviour in one's community.^19^ In a forensic mental health context, this might include past experiences of services, staff education and training, staff support, sufficient staffing, fair procedures, management/leadership style, structures for care plan meetings and an efficient forensic care treatment pathway/model.

## Evidence for the value of procedural justice?

Studies across the criminal justice system have found that when people experience procedurally fair interactions, broadly characterised by voice, dignity and respect, neutrality, and trust, they are more likely to have positive outcomes across a range of measures.

In a general mental health context, the MacArthur perceived coercion study found that perceptions of respectful inclusion in fair decision-making processes were associated with reduced experience of coercion. Using the same measure of procedural justice (the MacArthur Admission Experience Survey), McKenna et al.^14^ found that non-forensic psychiatric patients’ experiences of voice and validation (that their views were taken seriously) at admission to psychiatric settings significantly negatively predicted perceptions of coercion. They found the same relationships in a forensic setting.^20^ Following coercive experiences, patients have reported feeling that their human rights were violated, being disrespected, not heard by clinicians, unsatisfied with care and dehumanised; they have also felt less likely to consider staff as helpful, hindering the development of positive therapeutic relationships.^21–23^

Systematic reviews of research measuring procedural justice experiences in settings such as prisons, forensic hospitals, and courts have demonstrated associations between positive procedural justice experiences and greater satisfaction with legal decisions, less perceived coercion and less frequent use of illicit substances.^24,25^ In drug and mental health courts specifically, individual studies have found positive experiences of procedural justice linked with more frequent attendance in treatment sessions, improved relationships with and trust in staff, better quality of life, lower rates of depression and lower reoffending rates.^26–30^

Studies in prisons report positive procedural justice experiences have been associated with fewer rule violations and aggressive incidents, intrinsic motivation to cooperate with legal authorities and lower psychological burdens.^31–34^ A longitudinal study by Beijersbergen et al.^31^ found a temporally causal link between procedural justice experiences at three weeks and psychological burden at three months into a remand prison (i.e. jail) stay.

Though the evidence is indicative that better procedural justice experiences are linked with better outcomes, it should be noted that much procedural justice research in the criminal justice system is cross-sectional, using measures derived from models proposed in the 80s and 90s.^11^ These measures have been critiqued for lacking strong construct validity and conceptual clarity.^35,36^ Procedural justice scholars have recently argued for more inductive, qualitative investigations of experiences, aiming to expand our understanding of what procedural justice means for different people in different contexts.^37^

## Why do procedurally just experiences matter?

Tom Tyler, working with Lind^38^ and Blader^39^ has been influential in our understanding of why people care about being treated in a procedurally fair way. Drawing on social psychological perspectives, their work proposes that being treated in a procedurally fair way by people in positions of authority sends out a social signal about one's status.^40^ This signal communicates to a person not in a position of authority (the weaker party) that they deserve to be treated as a member of the social in-group, as somebody to whom dignity, respect and fair process ought to be afforded. People who do not experience procedural justice in their interactions are more likely to feel marginalised, less valued and excluded from the social in-group. For the weaker party, this is linked to identity: as Radburn and Stott^40^ put it ‘(…) the actions of the “powerful” upon the “subjugated” determine whether the individuals in question see themselves as a “respected citizen” or “valued” member of a superordinate social category, such as the “organization,” “community” or “nation state”’ (p. 4).

Consequently, those people with positive experiences are more likely to be committed to social norms, values and laws to maintain their position in the social in group. They are more likely to see these authorities and institutions as legitimate. Conversely, people with poor procedural justice experiences are less likely to subscribe to these values, follow the law and collaborate with legal authorities. Tyler's^41^ work stresses that procedurally fair treatment and the consequent feelings of group membership and belonging make individuals more likely to see their interlocutors as ‘legitimate’ and it is this legitimacy that is causally most closely linked to collaboration with authorities.

**Figure 1.**
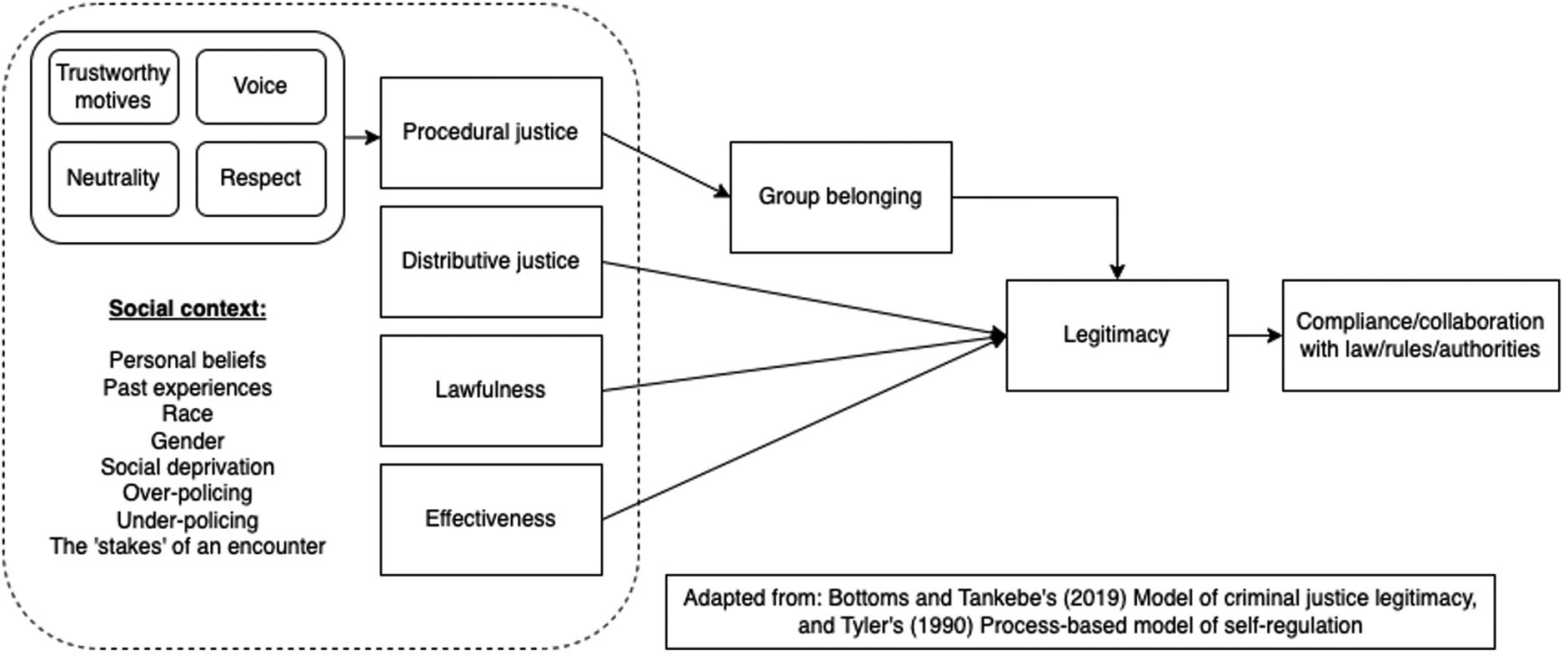
Overview of procedural justice theory based on Bottoms and Tankebe's^19^ model of criminal justice legitimacy and Tyler's^41^ process-based model of self-regulation.

To summarise, people's experiences of procedural justice are shaped by a range of individual and contextual factors. However, voice, dignity and respect, neutrality and trustworthiness are salient and recurrent principles of procedurally fair experiences. People who have interactions with power holders characterised by these principles are more likely to feel included, valued and committed to shared norms and cultural beliefs. This alignment promotes perceptions of legitimacy and a normative intention to collaborate with legal authorities and obey the law. This is depicted in Figure 1.

From a human rights perspective, these experiences have intrinsic value – reducing coercion and increasing agency and citizenship. From a critical standpoint, however, this does open possibilities for procedural justice to be used instrumentally by powerholders for the benefit of maintaining a social system and framework of laws designed to support that system. These concerns should not be overlooked, and the implementation of procedural justice principles should always be used in good faith, with patients’ best interests and individual dignity as sole motivators.

## Staff–patient interactions and procedural justice in forensic settings

In a forensic mental health context, procedural justice theory can help us to consider how interactions between patients and staff (as well as between staff) that involve decision-making can be experienced and improved. If findings from other contexts are transferable, exchanges that adhere to procedural justice principles are more likely to be seen as satisfactory, promote collaboration and engender feelings of belonging and worth in patients. As procedural justice experiences are fundamentally relational,^25,33^ the four principles can form a framework for recognising staff good practice, structuring staff training and informing reflective practice in forensic settings.

Qualitative literature on patient and staff interactions in forensic settings is helpful for understanding when and how procedural justice principles are/are not present in practice, and how procedural justice might be redefined in the forensic context. For example, Coffey's^42^ review of qualitative studies into patient experiences of care found that patients wanted staff to provide boundaries and supportive yet constructively challenging therapeutic assistance (not frequently considered in the procedural justice literature with the exception of Wittouck's work^18^) with clear communication of information (e.g. voice and respect). These accounts are sometimes characterised by feelings of powerlessness (e.g. voice), and confusion as to how the system works (e.g. neutrality, information); but are sometimes described as supportive (e.g. trustworthiness), with some patients requesting more frequent opportunities to talk with staff.

Forensic patients have described positive therapeutic relationships as characterised by feeling listened to (e.g. voice), receiving specific feedback (e.g. dignity and respect; neutrality), being involved in care planning (e.g. voice), and staff being genuinely interested in helping (e.g. trustworthiness).^43^ When reflecting on coercive measures, patient accounts highlight staff demeanour during restraint incidents as important, with individuals reporting feeling angry at staff who carried on conversations or laughed during restraint (demeanour is not commonly featured in accounts of procedural justice).^44^ For patients, rebuilding therapeutic relationships after restraint required open and clear communication (e.g. voice), time, demonstrating caring (e.g. trustworthy motives), use of appropriate body language, compassion in tone and asking patients what they want to do on a given day (e.g. respect and voice).^45^

The only qualitative study of procedural justice experiences in forensic settings we are aware of took place in Belgium.^18^ The author found that patients identified the following interrelated factors as important for procedurally just interactions: being in a caring environment, holding reciprocal dialogue, the quality of staff job performance, transparency, being treated holistically in a person-centred and solution-focused way, with firm but fair authority. These findings suggest that a forensic-specific conceptualisation of procedural justice includes principles identified in non-forensic mental health contexts and extending these.

Procedural justice principles are also relevant in police–patient interactions, experiences in custody suites, transfer from and between prison and hospital, court proceedings, tribunal hearings, in multidisciplinary team meetings, and so on. Procedural justice principles can be used in specific interactions, but they can additionally be used to inform treatment plans and future decisions. A review by Ray and Simpson^46^ found empirical support for patient voice and engagement in shared risk assessment, especially where risk assessment was paired with a structured intervention. As a means to maximise patient voice, McKenna et al.^15^ proposed greater adoption of Ulysses Contracts. These involve discussing with patients the measures they would like to take place when they are unwell and their decision-making abilities are impaired. Clinicians provide clear information to inform the discussion, engage in constructive dialogue and seriously listen to what a patient has to say. If a patient's decision-making becomes impaired, the Ulysses contract provides patients with evidence of what their previous preferences have been to maximise their decision-making capacity.

## Training, reflective practice and procedural justice

Staff training and reflective practices can be informed by procedural justice principles. Training on procedural justice principles rolled out in other criminal justice settings does appear to improve outcomes for recipients of training and their interlocutors (e.g. police and victims of crime). A systematic review looked at police training interventions that included at least one procedural justice principle.^47^ The review included 28 studies reporting 40 evaluations in a series of meta-analyses. They found that people who interacted with the police in the procedural justice intervention condition were significantly more likely to report positive experiences of procedural justice (OR = 1.47; 14 evaluations), compliance and cooperation (OR = 1.62; eight evaluations), and satisfaction and confidence (OR = 1.75; 29 evaluations). No significant meta-analytic finding was reported for perceptions of legitimacy as an outcome (OR = 1.58, *p* = 0.148; seven evaluations).

The Greater Manchester Police Procedural Justice Training Experiment is indicative of these interventions.^48^ Participants were taught to use names of members of the public, show empathy, develop rapport, give positive acknowledgements, reconsider use of certain words like ‘obviously’, signpost to support/next steps, say ‘no’ positively and agreeing when to move on after an incident. After three months, researcher observations of officer role-playing exercises were conducted. Officers in the treatment group (N = 339) were significantly more likely than controls (N = 237) to acknowledge victims' emotional state, empathise with the victim, give the victim a choice of options and use body matching. Between three and nine months after the training, victim perceptions of the overall quality of their interactions with the officers were significantly better than in the control group, though no differences were observed for victims’ willingness to cooperate, satisfaction with the way they felt treated or with service provided.

A recent exploratory evaluation found that reflective practice in forensic settings has positive effects for multidisciplinary working and individual staff-level factors, including confidence in one's role.^49^ Reflective practice could incorporate procedural justice principles, with specific interactions being reviewed in accordance with these. One suggestion is to create a debrief form. In the first column, the main principles of procedurally just interactions are listed. In the second column, staff completing the debrief sheet indicate whether they felt this principle was present, was somewhat present, or was not present in a particular interaction with a patient. In the third column, staff can reflect on why they think this principle was or was not present, what could be repeated or changed in a future interaction and how they can be supported by the service to perform in a procedurally just way. A final column asks whether there has been an improvement in this principle since a previous interaction. A similar form could be completed by patients and results discussed in a structured, supported setting.

Given the relational nature of procedural justice experiences, the above implicates a possible role for its implementation in the form of framing staff good practice, structuring staff training and informing reflective practice in forensic settings.

## Future directions for procedural justice in forensic mental health

Research into procedural justice in forensic mental health settings has begun. In Canada, Simpson et al.^50^ are conducting a longitudinal study using mixed methods to explore the extent to which ‘patient perceptions of coercion, fairness and legitimacy impact upon their level of treatment engagement, risk for adversity and progress in recovery’ (p. 1). In Belgium, Wittouck^18^ interviewed forensic mental health patients about their experiences of procedural justice and is now interviewing staff about self-legitimacy (the degree to which staff feel empowered, supported and justified in using power given to them). In the UK, Lawrence and Smothers are planning to introduce principles of procedural justice to a male medium secure ward and evaluate this (personal communication). Tomlin and colleagues will begin the ENGAGE study. This is a Grounded Theory investigation of staff and patient experiences of procedural justice across the care pathway to identify barriers and facilitators to improving and supporting patient engagement in care, specifically decision-making, jointly funded by the British Academy and the Leverhulme Trust.

There are exciting opportunities for future research. A key first task is to further explore how patients and staff experience procedural justice, which phenomena characterise positive procedural justice experiences and how these experiences vary across the care pathway. This work should draw on studies of non-forensic mental health populations but not be constrained by them, contextualising findings to the forensic mental health system.

Conceptualizing procedural justice in this way will allow for the operationalisation of the construct in this setting. With key principles articulated, measures of procedural justice and training programmes could be developed. A questionnaire measuring procedural justice experiences should draw on past efforts to capture the construct but be grounded in the above mentioned qualitative work. Staff training on embedding procedural justice principles in routine practice should be piloted, evaluated, revised and rolled out in randomised trials.

Ultimately, evidence attesting to the utility of procedural justice-informed practices is needed. This requires the longitudinal examination of the causal links between procedural justice and social and clinical outcomes. These might include recovery (e.g. DUNDRUM-4), therapeutic alliance (e.g. WAI), ward atmosphere (e.g. EssenCES), satisfaction with care (e.g. FSS), perceived coercion (e.g. MacArthur), overall psychological well-being (e.g. BSI), and post-discharge outcomes such as housing, employment offending and attending scheduled appointments. Experiences of procedural justice might be measured after important events and interactions, such as tribunal hearings or before and after staff training programmes and ward-level interventions.

Procedural justice presents another model for guiding interpersonal exchanges and another line of research inquiry. Staff and services are already stretched and expected to follow national, local, and evidence-based guidelines in myriad ways. However, if insights from procedural justice theory could be applied in forensic mental health settings to improve patient outcomes, staff–patient relationships and general conditions of work, it is a worthwhile endeavour.
